# Near vision data and near correction requirements from community eye health programmes in nine countries

**DOI:** 10.1038/s41433-023-02910-4

**Published:** 2024-01-22

**Authors:** Marzieh Katibeh, Elanor Watts, Michael Gichangi, Sergio Latorre-Arteaga, Nigel M. Bolster, Andrew Bastawrous

**Affiliations:** 1 Peek Vision, Berkhamsted, UK; 2 Department of Ophthalmology, Faculty of Medicine, Aalborg University Hospital, Aalborg, Denmark; 3 Tennent Institute of Ophthalmology, Glasgow, UK; 4 Directorate of Health Care Services, Ophthalmic Services Unit, Ministry of Health, Nairobi, Kenya; 5 Department of Optometry, Faculty of Health Sciences, Lúrio University, Nampula, Mozambique; 6 International Centre for Eye Health, London School of Hygiene and Tropical Medicine, London, UK

**Keywords:** Epidemiology, Health services, Refractive errors

## Abstract

**Background:**

Recent estimates of global prevalence of uncorrected presbyopia range from 510 to 826 million. There is a shortage of primary data regarding Near Visual Impairment (NVI) magnitude.

**Methods:**

Near visual acuity (NVA) and NVI data was collected from over 388,000 people aged 35 or over across 9 countries, within Community Eye Health programmes between January 2022 and June 2023. In Kenya (*n* = 34,328), dioptric power of required near correction was also recorded, and any association with age, gender or level of NVA was assessed via linear regression model.

**Results:**

146,801 of 388,939 people failed initial near vision screening (37.74%, 95% CI 37.59–37.89%), with significantly higher prevalence of NVI in Sub-Saharan Africa than South Asia. Of those with distance acuity 6/12 or better, 27.97% failed (95% CI 27.81–28.13%) with evidence of difference between genders (*p* < 0.001): 30.77% of women vs. 24.47% of men. The most commonly required dioptric powers of correction were +2.00D, +2.50D and +3.00D, and required power correlated with age and NVA.

**Conclusions:**

NVI remains common among Community Eye Health programme participants aged 35 and over. Data from large scale programmes such as these provide an opportunity to contribute to more accurate epidemiological estimates, and to guide future research, resource planning and intervention, ideally with improved standardisation of testing in the future.

## Introduction

Near visual impairment (NVI) is now defined by the World Health Organization (WHO) and ICD-11 as the inability to see N6 at 40 cm [1], compared to the previously commonly used N8 cutoff. NVI has been recognised as a priority in the field of eye health by the World Health Organization and the International Agency for the Prevention of Blindness [2]. The 2019 World Report on Vision [3] highlighted uncorrected presbyopia as one of the most prevalent causes of visual impairment, despite the existence of a cheap, effective solution: near correction spectacles, commonly referred to as reading glasses. It has been suggested that NVI is in fact damaging to more domains of quality of life and functioning than distance vision. [4] Despite this, many eye health programmes and surveys only measure distance visual acuity (DVA). Therefore, estimates of magnitude are based on more limited data than for DVA. A recent epidemiologic meta-analysis, Fricke et al. [5], used by the World Report on Vision [3], estimated that 826 million people suffered from unaddressed presbyopia worldwide in 2015. This was based on a combination of modelling based on expected amplitude of accommodation at each age, and myopia prevalence, and epidemiological population data from 25 studies with a combined sample size of 76,607; plus review of 43 studies of spectacle-correction coverage. A more recent report by the Vision Loss Expert Group used in Lancet Commission on Global Eye Health [6] estimated 510 million people worldwide to have uncorrected presbyopia in 2020. The latter was based primarily on nationally representative WHO SAGE and US NHANES surveys. As demonstrated in Table 1, the paucity of reliable data from many countries has necessitated extrapolation from only 17 countries in each estimate. We believe this represents a gap in primary evidence of NVI and uncorrected presbyopia prevalence. Indeed, both studies describe the need for further epidemiological evidence, to reduce reliance on modelling. The increasing use of digital data collection and management offers an opportunity to collect NVI prevalence data from large scale ongoing eye health programmes, to fill this gap.

**Table 1:** Currently accepted estimates of uncorrected presbyopia, and summary of source data.

Author	Fricke et al. (2018)	Vision Loss Expert Group (2020)
Title	Global prevalence of presbyopia and vision impairment from uncorrected presbyopia	Trends in prevalence of blindness and distance and near vision impairment over 30 years: an analysis for the Global Burden of Disease Study
Definition	Unaided near vision worse than N6 or N8 at 40 cm or customary working distance, excluding people with eye disease causing reduced near vision	Vision impairment from uncorrected presbyopia was defined as presenting near vision of worse than N6 or N8 at 40 cm when best-corrected distance visual acuity was 6/12 or better.
Global estimate of trends in total presbyopia magnitude	1,800,000,000 (for 2015), predicted to peak at 2,100,000,000 in 2030	Forecast magnitude to continue increasing
**Global estimate of uncorrected presbyopia magnitude**	**826,157,000 (for 2015)**	**510,000,000 (for 2020)**
Countries contributing to Source Data	Brazil, China, Eritrea, Fiji, India, Iran, Kenya, Mongolia, Nepal, Niger, Nigeria, Pakistan, Singapore, South Africa, Tanzania, Timor-Leste, USA	Australia, Brazil, China, Eritrea, Ghana, India, Kenya, Mexico, Nepal, Niger, Nigeria, Pakistan, Russia, South Africa, Tanzania, Trinidad and Tobago, USA
Years of Data Collection	1997–2014	1992–2017

Magnitude estimates are highlighted in bold.

## Methods

### Data sources

Eye health screening was introduced into pre-existing community and primary health services, including in primary health facilities (basic health units, rural health centres and dispensaries) or community health worker door-to-door visits. Data was collected from these eye health screening programmes in nine countries powered by Peek Vision and local and international stakeholders including CBM. These countries were: Ghana, Kenya, India, Nepal, Pakistan, South Africa, Tanzania, Uganda and Zimbabwe. The programmes included all age groups, however, data for presbyopia was acquired from those aged 40 and above, or 35 and above in two countries (Kenya and India). All available data gathered in live programmes from January 2022 to June 2023 was included.

### Data collection and measurement of near visual acuity/impairment

Community members were first sensitised via a range of means (including communication from community workers and social organisers, word of mouth, local radio announcements and posters) to attend the screening programmes. The screening was conducted either at the participants’ households via door-to-door screening programmes (*n* = 304,371, 78.26%), at local primary/community health facilities (*n* = 79,638, 20.48%) or at local schools (*n* = 4930, 1.27%). Those found to have distance visual impairment, near visual impairment, and/or other eye conditions at initial screening, were referred for further assessment to the secondary (triage) and/or tertiary levels. Primary eye health needs were addressed at the screening or triage levels, while people with further needs were referred to hospital ophthalmology services.

At initial screening stages, the method of identification of NVI varied between programmes. In some, this was carried out less formally by layman screeners, with assessment of reduced near vision causing functional limitation, using a chart or a locally relevant text, e.g. bible, newspaper or other printed media in the local language, at a comfortable reading distance (33–50 cm). The availability of spectacles at the time of examinations was recorded and the measurements were based on available correction at the time of screening, i.e. presenting acuity.

More precise NVA measurement was carried out at the triage step of the programme pathway. Participants who had been identified as having a problem during screening were examined by ophthalmic clinical officers (OCO) or equivalent cadre, and binocular NVA measured with a standard N notation reading chart. For those who would benefit from near correction spectacles, the dioptric power of spectacles required was measured by trained ophthalmic officers. In Kenya, data for dioptric power of required near correction was recorded (*n* = 34,328 participants).

Data was collected and transmitted digitally through a data collection smartphone app and Peek Admin online software (Peek Vision Ltd, Berkhamsted, UK).

### Inclusion and exclusion criteria

In comparison between countries and regions, participants with distance vision worse than 6/12 were excluded from near vision data analysis, to focus on those with NVI secondary to presbyopia rather than more general ocular problems such as cataract, which would not be solved by near correction spectacles. Participants were only included if within the age range at risk of presbyopia, as defined within each programme, either 40 and over, or 35 and over. There was no other exclusion of data based on demographics (gender or other sociodemographic factors). Where data collection was atypical among programmes, precluding comparison, e.g. monocular NVA, this was excluded.

### Data protection and ethics

Participation in the screening programme and further steps was voluntary. Data included in this study were anonymous with no participant-identifiable information, and processed according to the General Data Protection Regulation (European Union GDPR). As no individual identifiable data is included, and programme data is analysed on a regional level, individual consent was not required. Data was stored on a secure server, and transmission and processing were carried out via encrypted devices.

### Data analysis

Near visual impairment at the screening level was treated as a binary variable: pass or fail. Logistic regression was used to assess the association of demographic factors and distance VA with screening NV test result. At the triage stage, quantitative near visual acuity was measured in N units. Any association between the dioptric power of near glasses and age group, gender or initial level of visual impairment was assessed using a regression general linear model. Stata software version 14 (StataCorp, Texas, US) was used for data analysis. Graphs were created in Google Sheets (Google, California, US).

## Results

### Results of near vision testing

388,939 people aged 35 or over, from nine countries, underwent near vision testing during the vision screening programmes, mostly via door-to-door household screening (78.28%). 146,801 of these failed the initial near vision screening test (37.74%, 95% CI 37.59–37.89%). Among those screened, 306,832 people underwent both near and distance vision screening. The prevalence of failed NV screening among those with distance visual acuity 6/12 or better (*n* = 221,011) was 27.97% overall (95% CI 27.81–28.13%): 30.77% (95% CI 30.55–30.99%) among female participants vs. 24.47% (95% CI 24.24–24.70%) among males. This gender difference was statistically significant (*p* < 0.001). As shown in Table 2, increased age, poor distance VA, non-male gender, and sub-Saharan African location were associated with higher likelihood of failing near vision test during screening. Wearing or owning reading glasses was protective. After the screening stage, 91,197 people participated in the NV test at triage, of whom 79,162 (86.80%) failed the test.

**Table 2:** Logistic regression of factors influencing odds of failing near vision test at screening.

Independent factors	Sub groups	Number of people tested	Near VI at screening %	Adjusted Odds Ratio	*p* value	95% Confidence intervals	
Age groups	35–40	5645	12.83	Ref.			
	40–45	95,082	27.73	2.196	**<0.001**	2.023	2.383
	45–50	77,997	34.35	3.062	**<0.001**	2.820	3.323
	50–55	67,563	39.60	3.513	**<0.001**	3.235	3.815
	55–59	43,208	38.89	3.387	**<0.001**	3.115	3.682
	60–65	37,685	47.04	3.775	**<0.001**	3.471	4.105
	>65	61,759	51.23	3.215	**<0.001**	2.958	3.494
Gender	Male	170,669	34.00	Ref.			
	Female	218,229	40.67	1.354	**<0.001**	1.334	1.375
	Other	41	36.34	2.759	**0.003**	1.416	5.376
GBD region	South Asia	158,106	26.50	Ref.			
	Sub-Saharan Africa	230,610	45.44	1.196	**<0.001**	1.175	1.218
Distance VA screening	Pass	306,832	27.97	Ref.			
	Fail	74,294	75.95	6.726	**<0.001**	6.593	6.861
	Untested	7813	58.27	3.309	**<0.001**	3.154	3.473
Prior ownership of reading glasses	Wearing	33,405	17.82	Ref.			
	Own	79,903	15.69	1.004	0.833	0.968	1.041
	None	275,631	46.55	3.528	**<0.001**	3.415	3.645
Regression constant				0.039	**<0.001**	0.036	0.043

*P* values <0.05 are highlighted in bold.

**Fig. 1:**
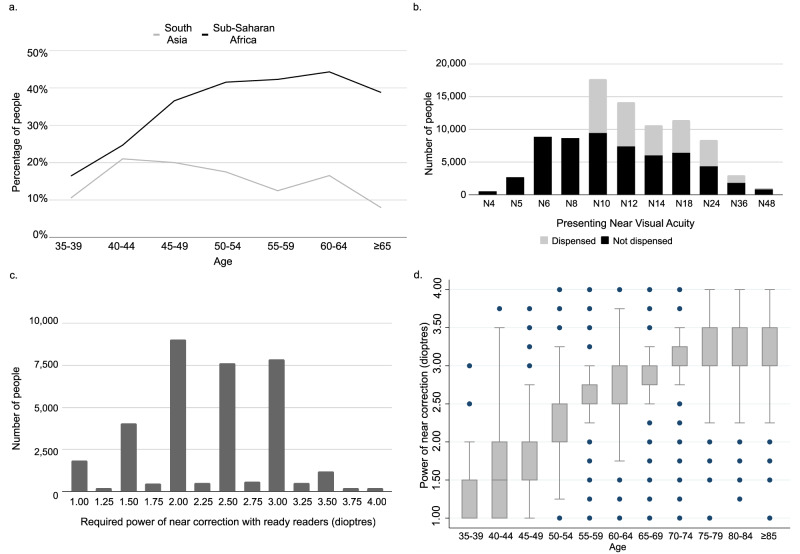
Near visual impairment prevalence, presenting near visual acuity, and required powers of near correction among programme participants. ** a** Percentage of participants with good distance vision (6/12 or better) who failed near vision test at screening, by age and GBDS region. **b** Distribution of presenting Near Visual Acuity at triage centres, among those who were and were not dispensed with near correction (readymade reading glasses). **c** Distribution of required powers of near correction (dioptres) according to assessment by trained eye care personnel. **d** Box plot demonstrating required powers of near correction (dioptres) by age.

The countries in the data set were located in two Global Burden of Disease Study (GBDS) super regions: South Asia (*n* = 158,106) and Sub-Saharan Africa (*n* = 230,610). Figure 1a shows the proportion of people who passed distance vision screening and failed the NV test, at the screening stage, by GBDS region. More details of age specific prevalence of NVI at screening is presented in Supplementary Table 1 and Supplementary Fig. 1.

### Test agreement

The agreement between informal screening of near vision and precise NVI/NVA testing at triage centres with eye care personnel is shown in Table 3. There was agreement between tests in the majority of people (69.78%) who underwent both tests (either passed at screening and triage, or failed at screening and triage). The remaining 30.22% had different results for informal local testing versus structured NVI testing (false positive: 7.8%, false negative: 22.34%; diagnostic accuracy: 69.78%, 95%CI: 69.4–70.16).

**Table 3:** Comparison of near vision test result at screening and formal NVI test result by trained eye care personnel at triage.

		Formal NVI test result by trained eye care personnel at triage		
		Pass	Fail	Total
Near vision test result at screening	Pass	3068	12,565	15,633
		5.46%	22.34%	27.80%
	Fail	4430	36,173	40,603
		7.88%	64.32%	72.20%
	Total	7498	48,738	56,236
		13.33%	86.67%	100%

### Ownership and provision of near vision correction/reading glasses

At the screening stage, 33,405 (8.59%) participants had reading glasses readily available, 79,903 (20.54%) owned reading glasses but did not have them available, and 275,631 (70.87%) did not own any. Among non-owners, 128,314 (46.55%) failed the NV test. After excluding those with DVA worse than 6/12, 36.15% (95% CI: 35.95–36.35, *n* = 74,270/205,449) of participants who did not own reading glasses failed NV screening, compared to only 11.38% (95% CI: 11.18–11.57) of owners.

Ready reader spectacles were provided to 30,142 (40.18%) people, equally provided for both genders (*p* value = 0.388). Figure 1b shows the distribution of near visual acuity and dispensing status of people who attended vision triage centres.

### Power of near correction

The dioptric powers of reading glasses used at the triage stage are shown in Fig. 1c. Of note, this data was exclusively collected in Kenya. The most common powers were +2.00D, +2.50 D and +3.00 D. Figure 1d shows the distribution of powers in different age groups. Supplementary Fig. 2 shows the distribution of prescribed power for ready readers by the level of presenting NV. Table 4 demonstrates how mean dioptric power of near correction at triage increased with age. Gender had no statistically significant effect on the required power of reading glasses. In those with no reading glasses (31,474 of 34,283 people with available power data), the level of NVA and DVA status were statistically associated with the required power of near correction glasses, but were weaker predictors than age.

**Table 4:** Linear regression of power of ready readers identified at triage (dioptres), demographics (age and gender) and presenting (uncorrected) near vision status among non-glasses-owners (*n*=31,474).

Independent factors	Sub groups	Number of people tested	Mean power	95% Confidence Intervals		Coefficient	*P* value	95% Confidence Intervals	
Age groups	40–44	3538	1.50	1.49	1.52	Ref			
	45–49	5745	1.83	1.82	1.84	0.32	**<0.001**	0.31	0.34
	50–54	7251	2.19	2.18	2.20	0.68	**<0.001**	0.66	0.69
	55–59	4399	2.51	2.50	2.52	0.99	**<0.001**	0.97	1.01
	60–64	4863	2.81	2.80	2.82	1.28	**<0.001**	1.27	1.30
	65–69	2410	2.90	2.88	2.91	1.36	**<0.001**	1.34	1.38
	70–74	2064	3.05	3.03	3.07	1.51	**<0.001**	1.49	1.54
	75–79	750	3.06	3.03	3.10	1.52	**<0.001**	1.49	1.56
	80–84	317	3.13	3.08	3.19	1.58	**<0.001**	1.54	1.63
	≥85	137	3.11	3.02	3.20	1.55	**<0.001**	1.48	1.62
Presenting near visual acuity (uncorrected, as non-glasses-wearers)	N10	9168	2.24	2.23	2.25	Ref			
	N12	7097	2.23	2.22	2.25	−0.01	0.072	−0.02	0.00
	N14	4739	2.33	2.31	2.35	0.02	**0.003**	0.01	0.04
	N18	5124	2.43	2.41	2.45	0.09	**<0.001**	0.08	0.11
	N24	3995	2.51	2.49	2.53	0.13	**<0.001**	0.11	0.14
	N36	1150	2.59	2.55	2.63	0.13	**<0.001**	0.10	0.15
	N48	201	2.72	2.63	2.81	0.15	**<0.001**	0.09	0.21
Distance visual acuity at screening	Pass (≥6/12)	23,165	2.25	2.24	2.26	Ref			
	Fail (<6/12)	7848	2.57	2.56	2.58	0.03	**<0.001**	0.01	0.04
	Untested	461	2.51	2.45	2.57	0.04	**0.022**	0.01	0.08
Gender	Male	12,037	2.46	2.45	2.47	Ref.			
	Female	19,437	2.26	2.25	2.27	−0.01	0.145	−0.02	0.00
	Regression constant					1.48	**<0.001**	1.46	1.50

Adj R-squared = 0.6005.

*P* values <0.05 are highlighted in bold.

## Discussion

There is a shortage of data regarding magnitude of presbyopia, and uncorrected presbyopia causing near vision impairment. Large scale eye health programmes provide an opportunity to contribute to population survey data, planning for eye health services, and estimation of required resources. A key strength of this study is the very large sample size (>380,000) from nine different countries from two GBDS regions with the highest levels of avoidable low vision. Another advantage is that the programmes were launched at the community level and referred people to the secondary level where it was possible to confirm test results with trained eye care personnel.

Unfortunately, to date, near vision testing has rarely been a primary objective of such programmes, and when included, as demonstrated here, it is often tested with less standardisation than distance acuity, using a range of charts and distances, a range of cutoffs (N8 rather than N6) or miscellaneous printed texts. This may be appropriate in some contexts, e.g. to test for ability performing a specific task relevant to the participant, considering local context, rather than following strict NVI definitions. It does however limit extrapolation of results of NVI prevalence. In this study, 78.26% of participants were recruited via door-to-door screening and the rest via community programmes. We hope that this sampling method will increase how representative the results are of the general population, however, as this data was collected within eye care programmes, not epidemiological survey, the reported prevalence of near vision test failure in the screening phase should be interpreted cautiously. Participation in screening was voluntary, and data was not collected regarding non-respondents, therefore sampling error and potential selection bias cannot be quantified. On one hand, those who were aware of a visual problem may have been more inclined to participate in vision testing; alternatively, people with lower levels of healthcare engagement, likely to have lower rates of presbyopia correction, may have declined. Given the moderately high proportion of false negatives compared to false positives (22.34% vs. 7.88%) (Table 3), the real prevalence of NVI may be higher than suggested in this paper. Despite the limitation that this informal (locally designed) testing poses, we believe that the programme data here still contributes useful evidence to the overall context of presbyopia and NVI and to service planning, as this data depicts the real situation which may be faced in community level programmes. Over a quarter of programme participants over 35 years with good distance vision, from a very large sample, were unable to see well at near: likely due to presbyopia. This magnitude of uncorrected NVI was found to disproportionately affect women, with a difference between genders of 6.30% (*p* < 0.001).

Table 2 shows some demographic factors were more associated with failing the near vision test at screening. Odds of failing consistently increased with age, female gender (Adjusted OR 1.35), Sub-Saharan location (Adjusted OR 1.19), poor distance vision and non-ownership of ready readers. Nevertheless, some of those who already owned near corrective glasses demonstrated residual unmet need, with 15–17% suffering from NVI despite glasses wear. Optimal assessment of effectiveness of presbyopia correction would consist of near eREC [7] (effective refractive error coverage) calculation. Available data did not allow this due to need for uncorrected, presenting, and best corrected visual acuities, which have previously not been routinely collected. Integration of these detailed data in intermittent Rapid Assessment of Avoidable Blindness (RAAB) surveys could fill this information gap.

More consistent are the results from the triage stage, and those regarding near correction requirements, which can be used to guide programme planning and provision of suitable ready readers. When prescribing reading glasses, different systems have been used across the world. Spectacle options include ‘ready readers’: pre-made spherical near correction glasses with the same power in both eyes’ lenses and no astigmatic correction; prescription reading glasses, personalised to an individual’s refractive requirements; or multifocal lenses, such as bifocals or varifocals/progressives. In some regions, reading glasses are readily available over the counter (as is common in many high income countries), while elsewhere they may only be dispensed by highly trained eye care professionals. This prohibits access to near correction in settings with a paucity of optometrists. A third distribution avenue is via general healthcare providers, including community healthcare workers. The WHO Afro Primary Eye Care Training manual [8] recommends using options +1.50 D, +2.00 D, +2.50 D and +3.00 D (in agreement with our findings in these programmes), starting with +1.50 D for everyone and working up in power until good near vision is achieved. Selection of power can also be based on age, or on presenting NVA (as in the THRIVE study [9]). Based on our evidence, both age and presenting NVA correlated with required power, however age was the strongest predictor. The results shown in Table 4 (mean power) and Fig. 1d (median power) could be used as a starting point for prescription of ready readers, to predict the power of spectacles by the characteristics of the participant: for example, 1.50 D for people aged 40–44 years, 2.00 D for 45–54 years, 2.50 D for 55–59 years and 3.00 D for over 60. These are similar but not identical to the ranges suggested by du Toit [10]. Our regression model (adjusted *R*^2^ = 0.6) provides an opportunity for future study, and further work based on this preliminary data will be used to validate a set of rules for prescribing reading glasses. In this large sample, the most commonly required correction powers were +2.00 D, +2.50 D and +3.00 D, suggesting that these powers of reading glasses should be prioritised when managing glasses supply.

Figure 1b showed that although only one third of participants were dispensed with ready readers at the triage sites, provision prioritised those who had more severe near visual impairments, fortunately with equality in dispensing among both men and women.

Where NVI and NVA testing is carried out, a standardised method should be used at all stages of testing, to allow for improved comparison of NVI prevalence data, for example with a recent validated digital near vision test [11], which can be easily incorporated into programme data collection protocols. Given the magnitude of unmet need due to uncorrected NVI, ongoing research should consist of the inclusion of near vision testing within comprehensive, integrated, person-centred eye care, as recommended within the World Report on Vision and Lancet Commission [3, 6].

### Supplementary information

Supplementary Figure 1

Supplementary Figure 2

Supplemental Table 1

## Data Availability

The data that support the findings of this study are not openly available due to reasons of sensitivity but are available from the corresponding author upon reasonable request. Data are located in controlled access data storage.
