# Supplementary figures and images for: Near vision data and near correction requirements from community eye health programmes in nine countries
Source: Eye (Lond). 2024 Jan 22;38(11):2150–5. doi: 10.1038/s41433-023-02910-4 (PMC11269744; doi:10.1038/s41433-023-02910-4)

Prevalence of uncorrected presbyopia, by age and country

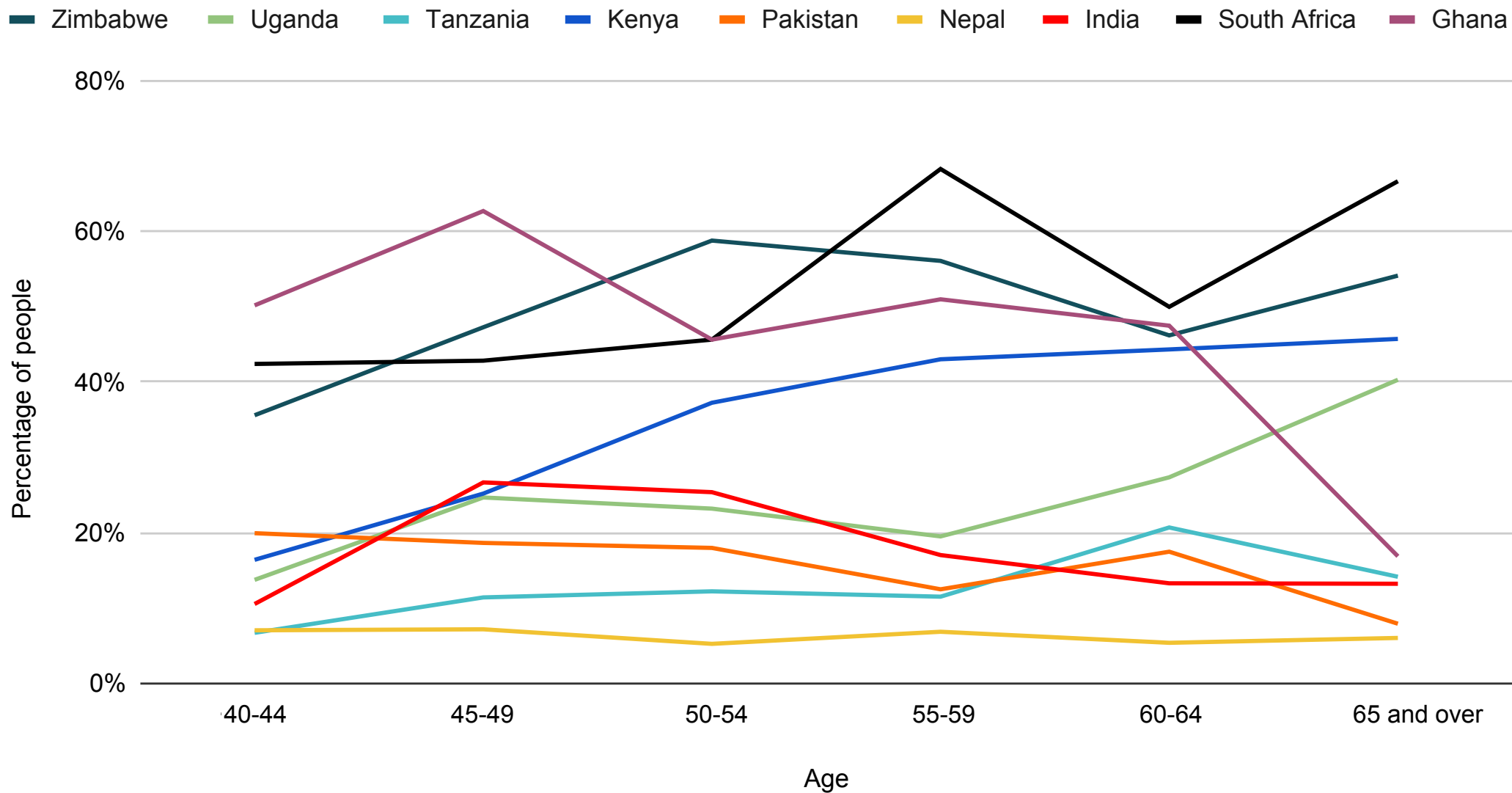

Supplement: Supplementary file 1 — Supplementary Figure 1 [file 41433_2023_2910_MOESM1_ESM.pdf]

Presenting near visual acuity by required near correction

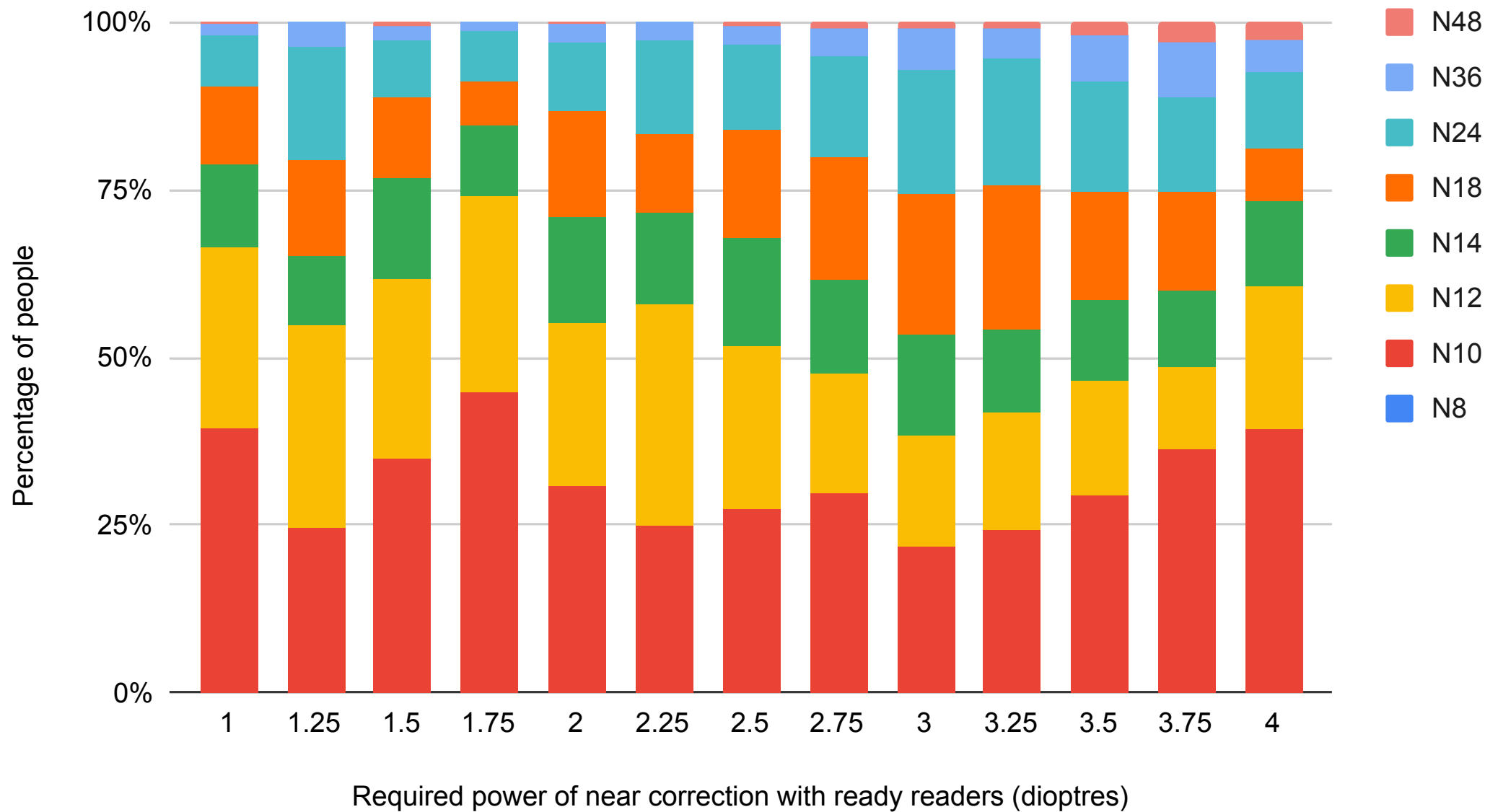

Supplement: Supplementary file 2 — Supplementary Figure 2 [file 41433_2023_2910_MOESM2_ESM.pdf]
